# Prevalence of depression and anxiety among patients with Behçet’s disease: a systematic review and meta-analysis

**DOI:** 10.1186/s42358-026-00591-7

**Published:** 2026-10-29

**Authors:** Golbarg Mehrpoor, Mohammad javad Amini, Arman Shafiee, Seyed Amirhossein Salehi, Reza Samiee, Gelareh Azarinoush

**Affiliations:** 1 Alborz University of Medical Sciences, Karaj, Islamic Republic of Iran; 2 Student Research Committee, School of Medicine, Alborz University of Medical Sciences, Karaj, Islamic Republic of Iran; 3 School of Medicine, Shahid Beheshti University of Medical Sciences, Tehran, Islamic Republic of Iran; 4 School of Medicine, Tehran University of Medical Sciences, Tehran, Islamic Republic of Iran; 5 Clinical research of development unit, Shahid Beheshti Hospital, Qom university of medical sciences, Qom, Islamic Republic of Iran

**Keywords:** Behcet's disease, Behçet’s, Depression, Anxiety, Meta-analysis

## Abstract

**Background:**

Behçet’s disease (BD) is a chronic multisystemic vasculitis associated with significant physical and psychological morbidity, including depression and anxiety. This systematic review and meta-analysis aimed to estimate the pooled prevalence of depression and anxiety among BD patients and to examine variations by assessment tools and geographic regions.

**Method:**

Following PRISMA guidelines, a comprehensive search of PubMed, EMBASE, and Scopus was conducted up to April 2025. Data were extracted and quality-assessed using standardized tools, and pooled prevalence rates were calculated via random-effects meta-analysis.

**Results:**

A total of 17 studies were included in the systematic review, of which 16 contributed to the meta-analysis of depression and 11 to the meta-analysis of anxiety among patients with BD. The results revealed a high pooled prevalence of depression at 47.44% (95% CI: 34.09–60.97%) and anxiety at 48.48% (95% CI: 35.40–61.67%), both with substantial heterogeneity (I² > 90%). Depression prevalence varied significantly by diagnostic instrument, with PHQ-9 and HADS reporting higher rates compared to DSM-IV and MINI. Geographic differences were marked, with Egypt showing the highest prevalence for both depression (88.83%) and anxiety (92.00%), while France reported the lowest prevalence for depression (5.00%) and Brazil reported the lowest for anxiety (29.31%). No publication bias was detected.

**Conclusion:**

The findings underscore a high burden of mental health comorbidities in BD, with pooled prevalence rates of **47.44%** for depression and **48.48%** for anxiety, reflecting significant psychological morbidity. Geographic disparities, suggest cultural, healthcare, or socioeconomic moderators requiring further investigation. These results advocate for integrating routine mental health screening into BD management to address unmet psychiatric needs in this population.

**Supplementary Information:**

The online version contains supplementary material available at https://doi.org/10.1186/s42358-026-00591-7.

## Introduction

Behçet’s disease (BD) is a type of vasculitis that affect arterial and venous vessels of all sizes, characterized by recurring oral ulcers, genital ulcers, and lesions affecting the eyes, skin, joints, nervous system, cardiovascular system, and gastrointestinal tract due to multisystemic inflammation [1]. BD is classified as a chronic, multisystem condition with unpredictable episodes of exacerbations and remissions [2]. While the etiopathogenesis of the disease is unclear, it is believed that genetic components, viral and bacterial influences, as well as both cellular and humoral immune responses play a role [3]. BD is particularly prevalent in nations along the historic “silk road,” an ancient trade route connecting China to the West. Turkey and Iran report the highest prevalence rates globally; in Turkey, estimates range from 80 to 420 per 100,000, while in Iran, the prevalence is approximately 80 per 100,000, with the most affected age group being those between 20 and 40 years old [4, 5]. There is no significant difference in the occurrence of BD between genders, but males tend to experience a more severe progression of the disease [6].

BD is associated with greater morbidity and mortality, especially in patients with ocular, central nervous system and vascular system involvement [7, 8]. BD can cause significant mental health issues, including depression and anxiety disorders, due to its chronic and recurring nature, the requirement for treatment with immunosuppressive and biological therapies, and various systemic symptoms [9, 10]. It can impact physical functioning and levels of activity [11]. Furthermore, the disease’s functional limitations and the associated stress can elevate anxiety levels. Chronic stress may impair immune system. Consequently, symptoms related to the disease may worsen in affected individuals [12].

Previous research has indicated that mental health issues such as anxiety and depression are prevalent among patients with Behçet’s disease (ranging from 8% to 50%), and 86% of these patients experience concurrent psychosomatic symptoms [13].A deeper understanding of the connection between BD and depression and anxiety is essential for creating and applying more effective treatment strategies for patients. Consequently, this systematic review seeks to explore the frequency of depression and anxiety in individuals affected by BD.

## Methods

### Study Protocol and Search Strategy

This systematic review was conducted in strict accordance with the Preferred Reporting Items for Systematic Reviews and Meta-Analyses (PRISMA) framework to ensure methodological rigor and enhance transparency throughout the research process [14].

### Search strategy

A systematic literature review was performed across electronic databases PubMed, EMBASE, and Scopus up to April 10, 2025. The search strategy incorporated keywords including “depression,” “anxiety,” and “Behcet’s disease,” along with their relevant synonyms and variations (e.g., “Behçet syndrome,” “depressive disorder,” “anxiety disorders”). No restrictions based on language were imposed during the search process. The full search strategy, including complete Boolean operators and database-specific syntax, is provided in the supplementary materials.

### Study selection

Three researchers independently conducted an initial assessment of the studies by analyzing article titles and abstracts to determine their eligibility for inclusion. When the title and abstract lacked sufficient detail to make an informed judgment, the full text of the article was reviewed comprehensively. Sufficient detail to make an informed judgment, the full text of the article was reviewed comprehensively. To ensure methodological rigor and relevance, the following inclusion criteria were applied:

1. Population: Studies involving patients with a confirmed diagnosis of Behçet’s disease (BD).
2. Outcomes: Studies reporting the prevalence of depression and/or anxiety.
3. Assessment Tools: Studies were required to use validated psychiatric assessment instruments. PHQ-9, HADS, BDI, and STAI were classified as screening tools designed to assess symptom burden, whereas DSM-IV and MINI were considered diagnostic instruments intended to identify clinically defined psychiatric disorders.
4. Study Design: Original research articles (e.g., cross-sectional, cohort studies) providing sufficient data for prevalence calculation.
5. Language and Publication: No restrictions were imposed on language or publication status.

Conversely, the following exclusion criteria were applied:

1. Review articles, case reports, editorials, and animal-based research.
2. Studies that failed to satisfy the established inclusion criteria or lacked sufficient data for extraction.
3. Duplicate publications or overlapping data sets.

### Data extraction

Three reviewers independently performed data extraction using a standardized template to collect relevant study information. The extracted data encompassed key details, including author names, publication year, country, Study Type, Depression/Anxiety assessment scale, participant demographics, and sample size.

To ensure accuracy, discrepancies in data interpretation were resolved through consensus-based discussions among the reviewers.

### Quality assessment

The methodological rigor of all included studies was evaluated using the Joanna Briggs Institute (JBI) Critical Appraisal Checklist for Prevalence Studies (CACPSS), a nine-item instrument with four response categories (yes, no, unclear, not applicable) [15]. Studies achieving a cumulative score of 8 or higher met the predefined quality thresholds and were retained for inclusion in the systematic synthesis and meta-analysis. To ensure objectivity, three authors independently evaluated study quality and bias risks, with unresolved discrepancies adjudicated through consultation with a fourth reviewer.

### Data analysis

We performed a meta-analysis using a proportion random effects model to estimate the prevalence rates of depression, and anxiety. Due to the variability in study populations and methodologies, we opted for a random effects model to pool prevalence estimates, reporting proportions along with their 95% confidence intervals (95% CI). Heterogeneity among studies was assessed using the Cochrane Q-test and the I² statistic, with I² values exceeding 75% indicating substantial heterogeneity. The statistical significance of differences between subgroups was assessed using the test for subgroup differences (Q-test), with a two-sided P value < 0.05 considered statistically significant. Publication bias was evaluated through funnel plot if outcomes had at least 10 effect sizes. All statistical analyses and visualizations were conducted using STATA17.

## Result

### Study selection and characteristics

In this systematic review, studies were included if they involved patients diagnosed with Behçet’s disease and assessed depression and anxiety using validated, scientifically accepted methods at baseline. Case reports were excluded to maintain methodological rigor. There were no restrictions regarding study location or publication language. Only original research articles employing well-defined diagnostic criteria for both Behçet’s disease and psychiatric evaluations were considered eligible for inclusion.

**Fig. 1:**
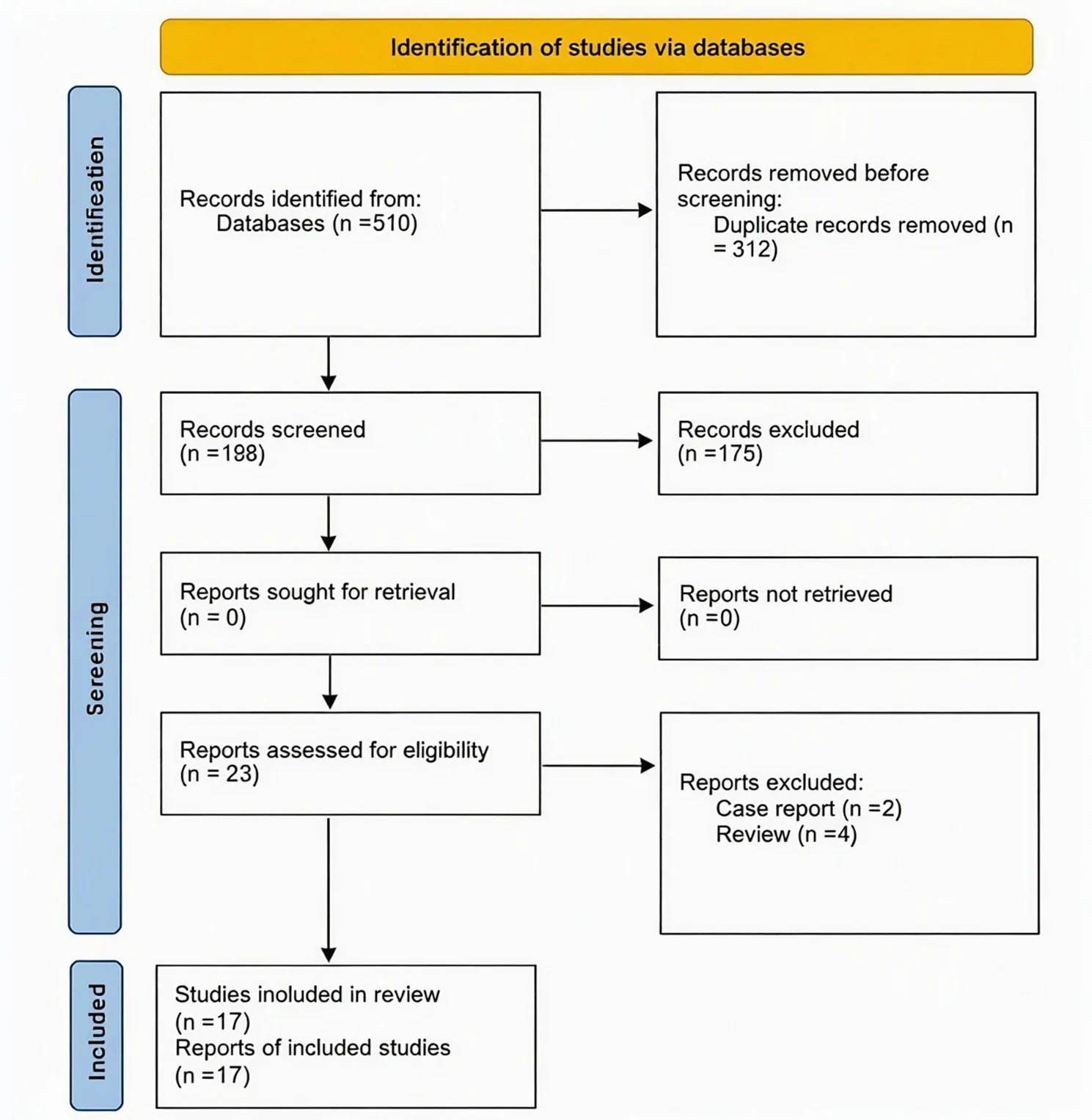
PRISMA flow chart

A systematic search conducted across PubMed, Embase, and Scopus databases initially identified 510 records. Following duplicate removal, 198 studies underwent screening, with 17 meeting the predefined inclusion criteria and being retained for final analysis. The study selection process is detailed in the PRISMA flowchart (Fig. 1).

From the 17 included studies, a total of 16 studies examining depression and 11 studies assessing anxiety in patients with Behçet’s disease, as several studies reported on both outcomes [10, 16–31]. These studies utilized a variety of validated assessment tools, including the Beck Depression Inventory (BDI), Diagnostic and Statistical Manual of Mental Disorders (DSM-IV), Hospital Anxiety and Depression Scale (HADS), Mini-International Neuropsychiatric Interview (MINI), and Patient Health Questionnaire-9 (PHQ-9) for depression, as well as the Beck Anxiety Inventory (BAI), DSM-IV, Generalized Anxiety Disorder-7 (GAD-7), HADS, MINI, and State-Trait Anxiety Inventory (STAI) for anxiety. See Table 1 for further elaboration.

**Table 1:** Summary of included studies

First Author / Year	Country	Study Design	Age (Mean/Median)	Sample Size (*n*)	Sex (M/F)	Assessment Tool(s)	Prevalence (*n*/*N*)
Oklar et al. 2024	Turkey	Retrospective cohort	37.24 ± 9.03	55	39/16	Depression: BDI; Anxiety: BAI	Depression: 36/55 (65.5%); Anxiety: 45/55 (81.8%)
Murray et al. 2021	UK	Cross-sectional	43.3 ± 13.97	103	27/76	Depression: PHQ-9; Anxiety: GAD-7	Depression: 70/107 (68.0%); Anxiety: 56/107 (54.4%)*
Teixeira et al. 2025	Brazil	Cross-sectional	46.1	58	31/27	Depression/Anxiety: HDRS	Depression: 25/58 (43.1%); Anxiety: 17/58 (29.3%)
Zayed et al. 2011	Egypt	Cross-sectional	33.8 ± 8.69	25	19/6	Depression: HDRS; Anxiety: HARS	Depression: 24/25 (96.0%); Anxiety: 23/25 (92.0%)
Yazmalar et al. 2014	Turkey	Cross-sectional	35.77 ± 8.84	112	79/33	Depression/Anxiety: HADS	Depression: 69/112 (61.6%); Anxiety: 44/112 (39.3%)
Urbain et al. 2023	France	Cross-sectional	38 (30–45.5)	20	16/4	Depression/Anxiety: MINI	Depression: 1/20 (5.0%); Anxiety: 9/20 (45.0%)
Saur et al. 2021	Germany	Prospective cohort	40.5 (18–64)	106	46/60	Depression: BDI	Depression: 39/106 (36.8%); Anxiety: NR
Pitaro Alter et al. 2022	Israel	Cross-sectional	48.9	873	461/412	Depression: BDI	Depression: 82/873 (9.4%); Anxiety: NR
Dursun et al. 2007	Turkey	Cross-sectional	37.4 ± 10.8	73	30/43	Depression/Anxiety: DSM-IV	Depression: 13/73 (17.8%); Anxiety: 26/73 (35.6%)
Koçak et al. 2009	Turkey	Cross-sectional	33.4 ± 7.5	71	0/71	Depression: BDI	Depression: 41/71 (57.7%); Anxiety: NR
Kalenderoglu et al. 2019	Turkey	Cross-sectional	37.58 ± 10.50	48	28/20†	Depression: DSM-IV	Depression: 21/48 (43.8%); Anxiety: NR
Ilhan et al. 2016	Turkey	Cross-sectional	39.4 ± 10	123	52/71†	Depression/Anxiety: HADS	Depression: 54/123 (43.9%); Anxiety: 35/123 (28.5%)
Hiz et al. 2011	Turkey	Cross-sectional	33.7 ± 7.2	42	42/0	Depression: BDI	Depression: 42/42 (100%)‡; Anxiety: NR
Gül et al. 2012	Turkey	Cross-sectional	35.8 ± 8.9	50	24/26†	Depression: HDRS	Depression: 29/50 (58.0%); Anxiety: NR
Sandikci et al. 2018	Turkey	Cross-sectional	37.0 (18–66)	155	86/69†	Depression/Anxiety: HADS	Depression: 63/155 (40.6%); Anxiety: 67/155 (43.2%)
Eser-Öztürk et al. 2020	Turkey	Cross-sectional	34.76 ± 11.14	58	48/10†	Depression: BDI; Anxiety: STAI-I/II	Depression: 18/58 (31.0%); Anxiety: 34/58 (58.6%)
Kabul et al. 2023	Turkey	Cross-sectional	43 ± 11.96	50	23/27†	Anxiety: BAI	Depression: NR; Anxiety: Low 36/50 (72.0%), Moderate 9/50 (18.0%), High 5/50 (10.0%)

*Abbreviations: BD = Behçet’s disease; CNS = central nervous system; NA = not applicable; NR = not reported; UK = United Kingdom; BDI = Beck Depression Inventory; PHQ-9 = Patient Health Questionnaire-9; HADS = Hospital Anxiety and Depression Scale; MINI = Mini-International Neuropsychiatric Interview; DSM-IV = Diagnostic and Statistical Manual of Mental Disorders, Fourth Edition; HDRS = Hamilton Depression Rating Scale; HARS = Hamilton Anxiety Rating Scale; GAD-7 = Generalized Anxiety Disorder-7; STAI = State-Trait Anxiety Inventory; BAI = Beck Anxiety Inventory

### Prevalence of depression in behçet’s disease

**Fig. 2:**
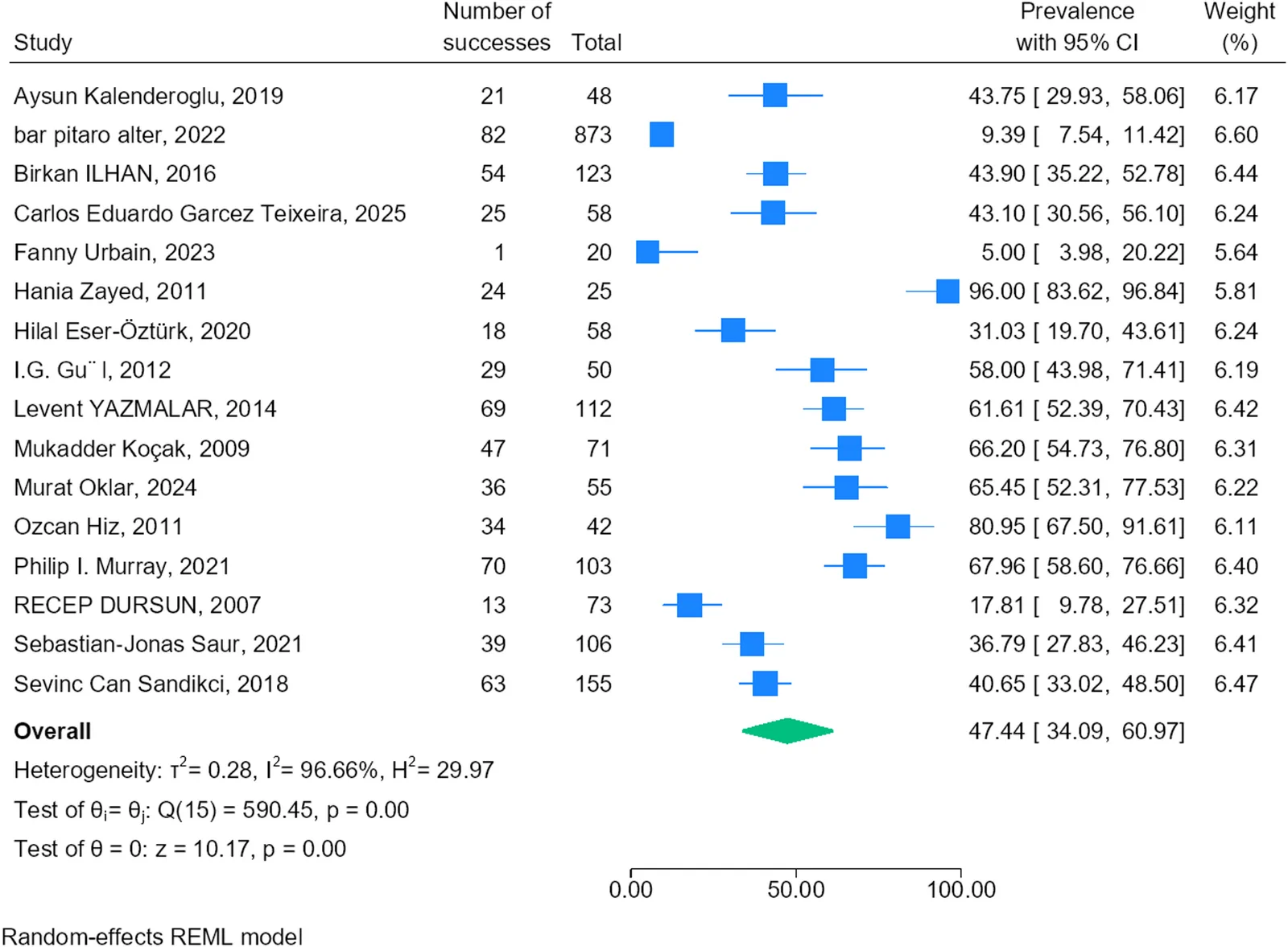
Prevalence of depression in people with Behcet disease

The pooled prevalence of depression among BD patients was estimated at 47.44% (95% CI: 34.09–60.97%), with significant heterogeneity across studies (I² = 96.66%) (Fig. 2), suggesting substantial variability in reported prevalence rates. The prevalence of depression varied depending on the screening instrument used. The highest prevalence was reported with PHQ-9 (67.96% [58.60–76.66%]) and HADS (60.77% [39.37–80.26%]), whereas studies utilizing the DSM-IV criteria reported a lower prevalence (29.65% [8.18–57.21%]). The MINI, which was applied in only one study, reported the lowest prevalence (5.00% [0.00–20.22%]). A test of group differences indicated that the variations between assessment tools were statistically significant (Q_b = 37.88, *p* < 0.001), highlighting the impact of measurement method on prevalence estimates (Supp fig. 1). In subgroup analysis according to the assessment method, the pooled prevalence of depression was significantly lower among studies using diagnostic tools (21.03%, 95% CI: 4.06–45.45; *n* = 3) than among those using screening tools (53.57%, 95% CI: 39.75–67.12; *n* = 13), with a significant difference between subgroups (Q_b (1) = 5.13, *P* = 0.02). There was considerable variation in depression prevalence across different countries. Studies conducted in Egypt reported the highest prevalence (88.83% [71.07–99.21%]), whereas those from France reported the lowest (5.00% [0.00–20.22%]). The prevalence in Turkey, which contributed the largest number of studies (*n* = 9), was 47.24% [36.12–58.50%], aligning closely with the overall estimate. A statistically significant difference was observed between countries (Q_b = 291.04, *p* < 0.001), suggesting that geographic and potentially cultural or healthcare-related factors may influence the observed prevalence rates. Assessment of publication bias using Egger’s test and funnel plot asymmetry did not suggest risk of publication bias.

### Prevalence of anxiety in behçet’s disease

**Fig. 3:**
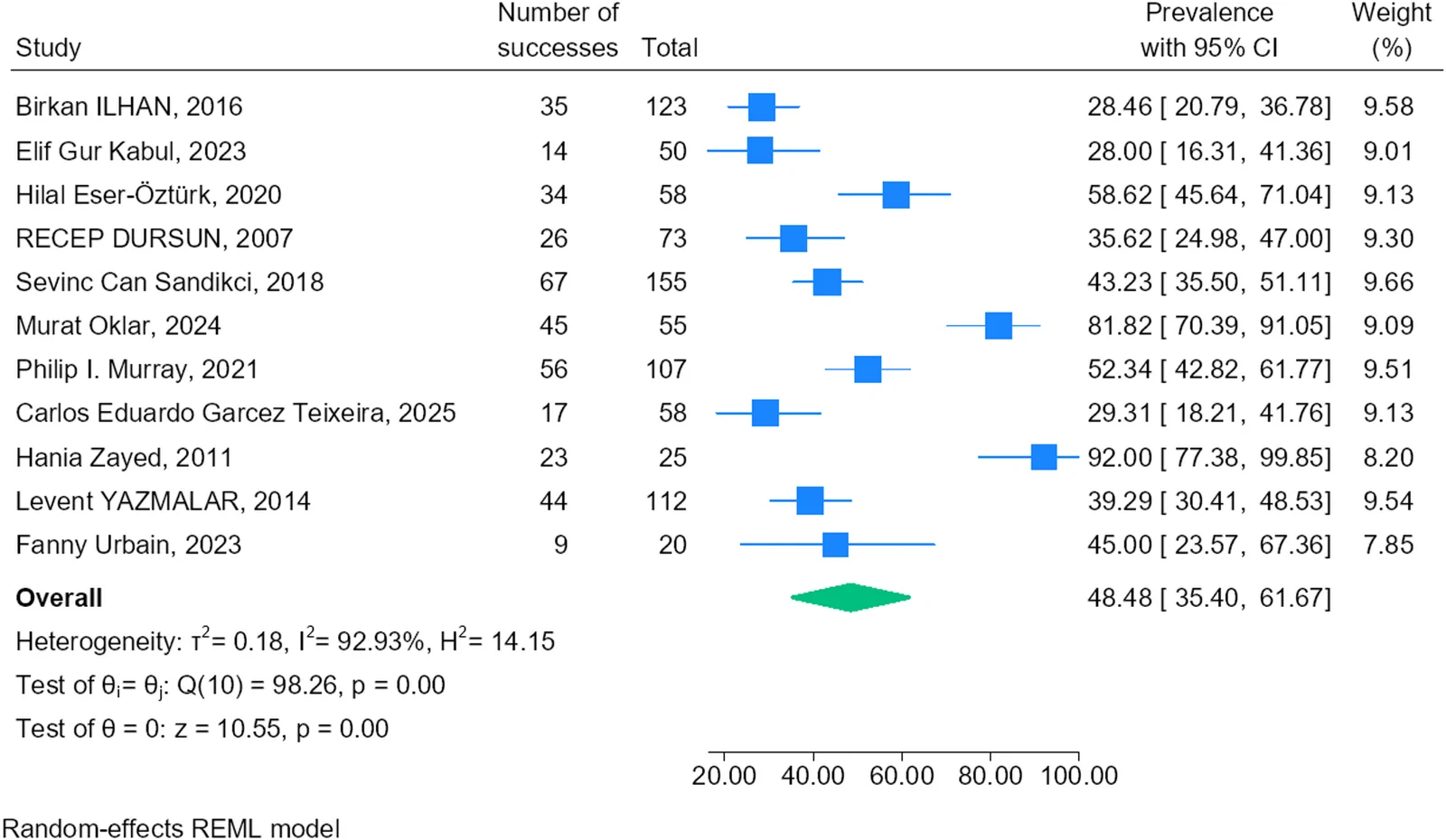
Prevalence of anxiety in people with Behcet disease

**Fig. 4:**
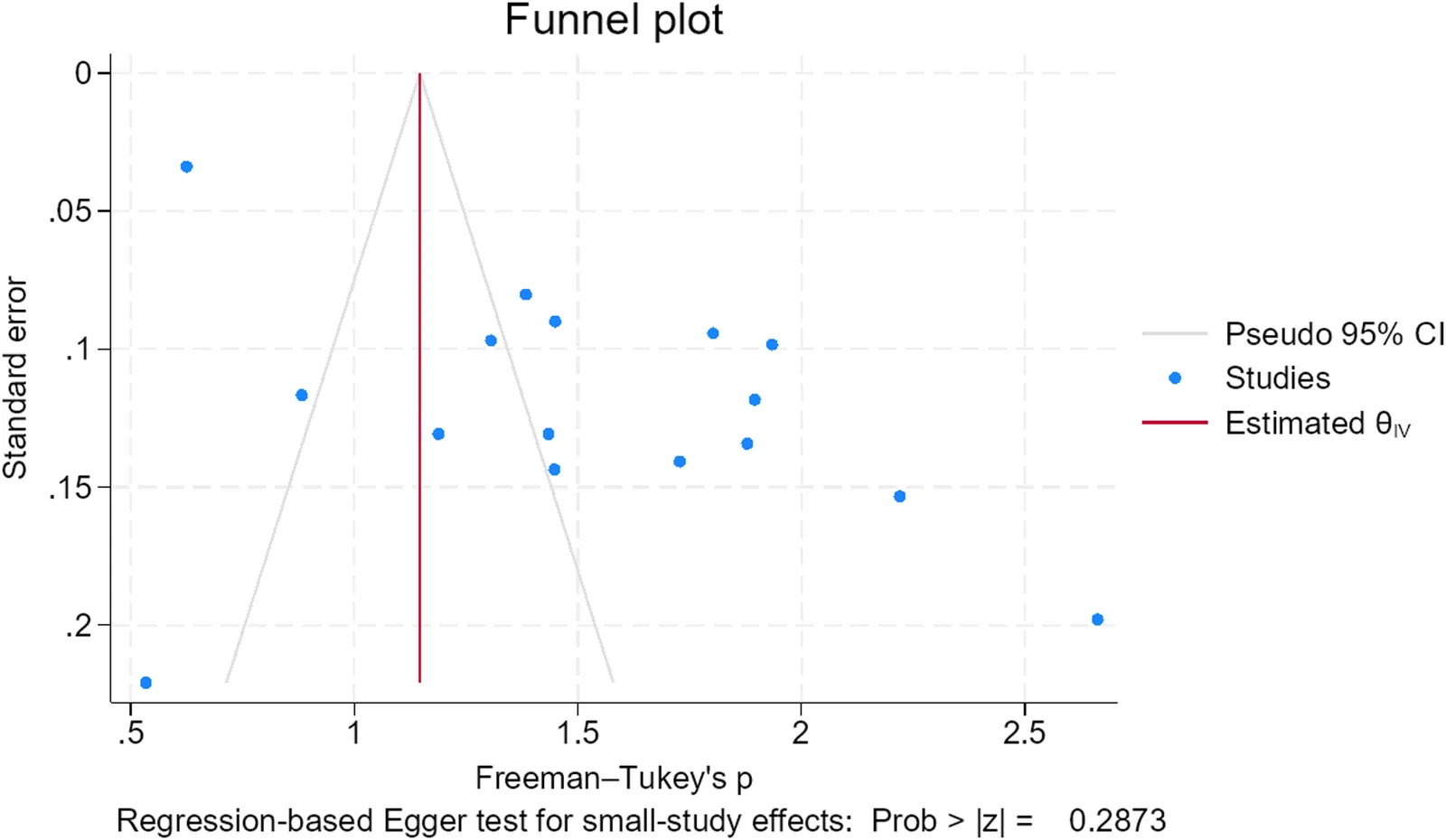
Funnel plot assessment for depression

**Fig. 5:**
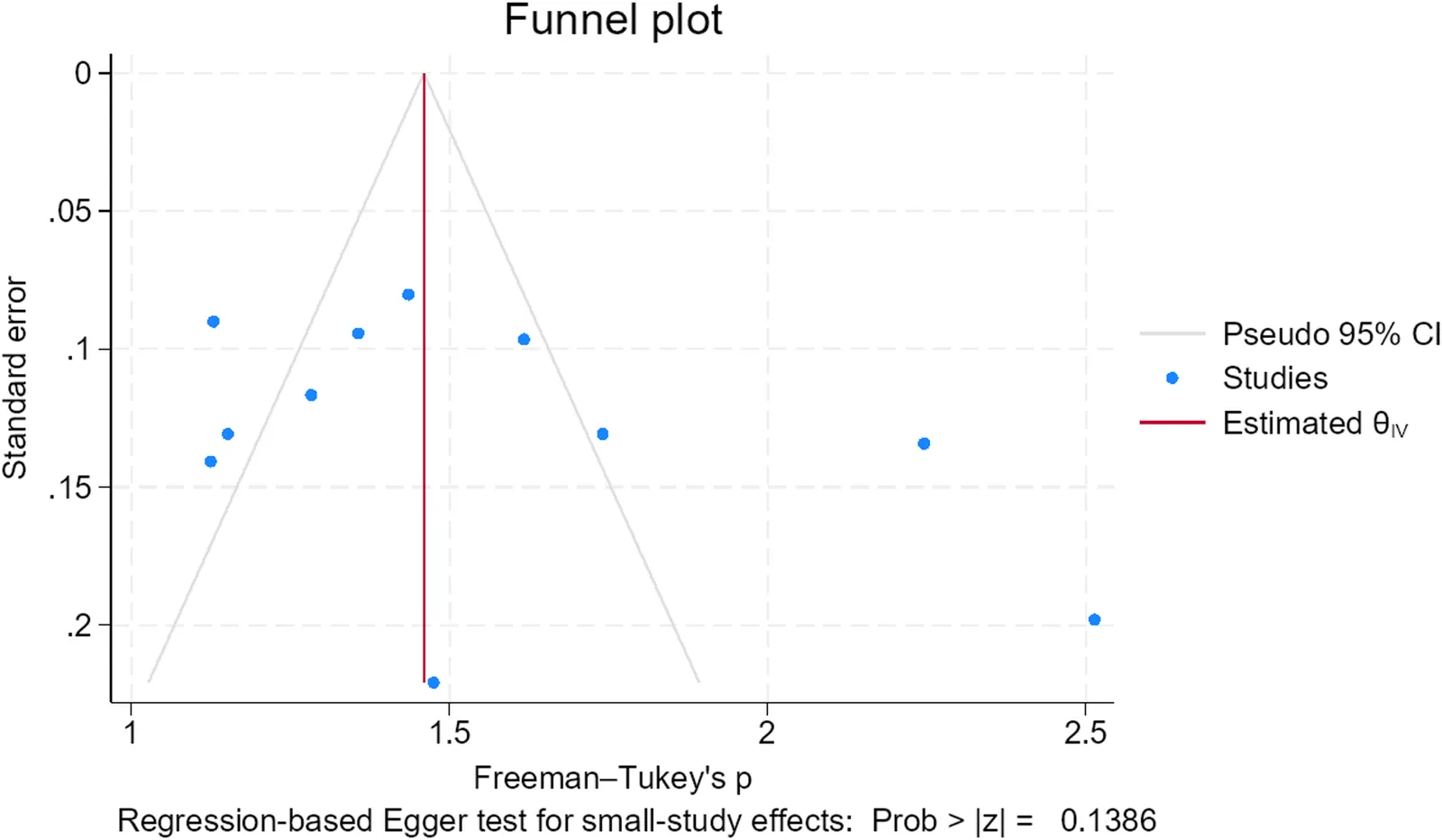
Funnel plot assessment for anxiety

The pooled prevalence of anxiety among BD patients was 48.48% (95% CI: 35.40–61.67%), with high heterogeneity (I² = 92.93%) (Fig. 3), indicating notable differences across studies. Anxiety prevalence estimates also varied depending on the assessment tool used. The highest prevalence was reported using STAI (58.62% [45.64–71.04%]), followed by BAI (55.87% [7.60–97.68%]) and GAD-7 (52.34% [42.82–61.77%]), while studies utilizing DSM-IV (35.62% [24.98–47.00%]) reported lower prevalence rates. However, the test for differences between assessment tools did not reach statistical significance (Q_b = 8.04, *p* = 0.15), suggesting that variations in reported anxiety prevalence were less influenced by the choice of instrument compared to depression (Supp fig. 2). The pooled prevalence of anxiety was 37.45% (95% CI: 27.67–47.75%) among studies using diagnostic tools and 50.39% (95% CI: 34.66–66.09%) among those using screening tools. The difference between these subgroups was not statistically significant (Qb(1) = 1.77, *p* = 0.18). Similar to depression, there was significant variability in the prevalence of anxiety across different countries. The highest prevalence was reported in Egypt (92.00% [77.38–99.85%]), while lower estimates were observed in Brazil (29.31% [18.21–41.76%]) and France (45.00% [23.57–67.36%]). Studies from Turkey, which accounted for the majority of included studies (*n* = 7), reported an anxiety prevalence of 44.86% [30.68–59.47%]. The observed differences between countries were statistically significant (Q_b = 33.83, *p* < 0.001). Assessment of publication bias using Egger’s test and funnel plot asymmetry did not suggest risk of publication bias (Fig. 4 and Fig. 5).

### Quality assessment

All studies were assessed according to the Joanna Briggs Institute (JBI) Critical Appraisal Checklist for Prevalence Studies and met the appropriate criteria for inclusion in the study, the relevant table of which is available in the supplementary file.

## Discussion

Patients with BD often experience a range of psychological symptoms, most notably depression and anxiety. These mental health challenges are frequently overlooked but can significantly affect quality of life and disease management. Fatigue, pain, physical disfigurement, and social isolation may further exacerbate psychological distress. Studies have shown that a substantial proportion of BD patients suffer from symptoms of anxiety, depression, and somatization, which may worsen with disease severity and duration [32–34]. Moreover, the bidirectional relationship between inflammation and mental health suggests that psychological stress may also influence disease activity, highlighting the importance of integrated care that addresses both physical and emotional well-being.

BD is a systemic vasculitis with unknown etiology. The disease generally manifests itself with recurrent oral and genital ulcers, skin lesions, and uveitis and sometimes there is neural system involvement better known as neuro- Behçet’s disease [35]. It can also affect the joints, blood vessels, gastrointestinal tract, and central nervous system [36]. The exact cause of BD remains unknown, but it is believed to involve a combination of genetic, environmental, and immunological factors [37]. Moreover, studies have highlighted a high prevalence of psychiatric comorbidities, such as depression and anxiety, in BD patients, further complicating disease management [19, 28]. Since BD is a chronic condition that needs to be dealt with on a daily basis, the idea that whether it can affect one’s mental health emerges [20].

This meta-analysis examined the prevalence of depression and anxiety in patients with Behçet’s disease (BD), analyzing 16 studies on depression and 11 on anxiety. The pooled prevalence of depression was 47.44% (95% CI: 34.09–60.97%), with significant heterogeneity (I² = 96.66%). Prevalence varied by assessment tool, with PHQ-9 and HADS reporting the highest rates (67.96% and 60.77%, respectively), while DSM-IV and MINI reported lower rates. Geographic differences were notable, with Egypt showing the highest depression prevalence (88.83%) and France the lowest (5.00%). Anxiety prevalence was 48.48% (95% CI: 35.40–61.67%), with high heterogeneity (I² = 92.93%). STAI reported the highest anxiety prevalence (58.62%), while DSM-IV reported lower rates (35.62%). Egypt also had the highest anxiety prevalence (92.00%), with Brazil and France reporting lower rates. Geographic and cultural factors significantly influenced prevalence rates. No publication bias was detected. Overall, depression and anxiety are highly prevalent in BD patients, with variability influenced by assessment tools and geographic location.

Recent meta-analytic findings indicate that 17.9% of rheumatoid arthritis (RA) patients meet criteria for a current depressive disorder, with a lifetime prevalence of 32.4%. Anxiety disorders affect 13.5% currently and 22.2% over a lifetime. Major Depressive Disorder is the most common, and prevalence is higher when assessed via self-report scales. Compared to the general population, RA patients face a significantly elevated risk, highlighting the need for integrated mental health care [38]. Depression affects 11–39% of SLE patients and may be the first symptom of the disease. Anxiety disorders are also common, with prevalence reaching up to 24%. Studies show SLE patients have a fourfold higher risk of depression and twice the rate of anxiety compared to the general population. Psychiatric symptoms often go undiagnosed, despite significantly impacting quality of life [39]. These findings suggest that BD imposes a psychological burden similar to or greater than that of other well-recognized autoimmune disorders. Given that BD often affects younger individuals and involves unpredictable flares across multiple organ systems—including neurological and mucocutaneous manifestations—the high rates of mental health comorbidity are not surprising but warrant greater clinical attention.

When compared to general population estimates, the burden of depression and anxiety in Behçet’s disease (BD) patients is markedly elevated. In Egypt, national surveys report a prevalence of depression of 9.5% and anxiety of 8.8 [40, 41]. In France, the prevalence of depression is 11.5% and anxiety disorders affect 10.6% of the population [42].In contrast, our meta-analysis found pooled prevalence rates of 47.4% for depression and 48.5% for anxiety among BD patients.

The disparity in depression prevalence between Egypt (88.8%) and France (5.0%) likely stems from methodological and contextual factors. The French study used the MINI, a diagnostic interview with high specificity that may underestimate prevalence, while the Egyptian study used the HDRS, which is more sensitive to mood symptoms [20, 21]. Egypt faces limited mental health resources and high stigma, increasing unmet needs [40], whereas France’s universal healthcare and integrated psychiatric services support better mental health outcomes [42].These differences in assessment, access to care, and sociocultural context likely contribute to the observed contrast [43].

It is important to note that the observed geographic variation in depression prevalence may be partly attributable to differences in assessment methodologies. For instance, the lowest depression rate was reported in France (5.00%), where the Mini-International Neuropsychiatric Interview (MINI) was used. MINI is a structured diagnostic tool with high specificity, designed to confirm DSM-IV criteria-defined disorders, and may therefore underestimate prevalence by excluding individuals with subsyndromal or mild symptoms. In contrast, higher rates in countries like Egypt (88.83%) were identified using the HDRS, a clinician-rated scale sensitive to a broader range of depressive symptoms. Similarly, PHQ-9 and HADS, which are screening tools with higher sensitivity, consistently yielded higher prevalence estimates across studies. Given that our subgroup analysis revealed significant differences in prevalence by assessment tool (Q_b = 37.88, *p* < 0.001), we caution that cross-national comparisons may reflect methodological heterogeneity as much as—or more than—true geographic or cultural differences.

In a systematic review and meta-analysis by Masoumi et al. [44] the impact of depression on the health-related quality of life (HRQOL) in patients with Behçet’s disease (BD) was investigated. Firstly, the article highlights that depression is a common comorbidity in BD patients, with prevalence rates ranging from 20% to 74% depending on the population studied. Moreover they stated that depression can act as a deteriorating factor for BD. Interestingly, the study found a positive correlation between depression and physical HRQOL, meaning that as depression scores increased, physical HRQOL scores also increased slightly (0.26 for each unit increase in depression). This counterintuitive finding may be due to the complex interplay between physical symptoms and psychological distress in BD patients. The study also highlights the importance of psychological support and depression management in improving the overall quality of life for BD patients.

Taner et al. [45] compared depression and anxiety levels between patients with Behçet’s disease (BD) and psoriasis, involving 112 BD patients and 95 psoriasis patients. For the purpose of their study they used Beck’s Depression Inventory (BDI), Beck’s Anxiety Inventory [20], Automatic Thoughts Questionnaire (ATQ), and Beck’s Hopelessness Scale (BHS) for assessment of the patients. The results indicated that all the compared measures were significantly more prevalent in the BD population. For instance, depression was witnessed in 45.5% of BD patients, while only 4.2% of psoriasis patients had depression. The study found a strong correlation between disease duration and depression, with longer disease duration significantly increasing depressive symptoms. The risk of depression was nearly 12 times higher in BD patients with a disease duration exceeding three years. Concerning anxiety, the results showed that this condition was more common among the youth, while depression became more prominent in older age groups.

According to the review article by Fisher [46], depression and anxiety are the most well-defined psychological disorders in Behçet’s disease and neuro-Behçet’s disease (NBD) patients. According to research, BD patients have a high rate of mental disorders including depression and anxiety in comparison to healthy controls, with depression being the most studied psychological disorder. The rates of depression in BD patients are consistently higher, with reports of depression in up to 60% of neuro-Behçet’s patients, especially in those with cognitive impairment. Anxiety is also very common - it is frequently comorbid with depression, and is linked with disease factors, including fatigue and arthritis. Both conditions are associated with disease activity, disease duration, and functional impairment, including erectile dysfunction in men. Broad-range psychological assessments indicate that > 40% of BD patients fulfill criteria for at least one mental health disorder (e.g., major depression and anxiety disorders such as generalized anxiety disorder, specific phobia). BD patients score in the mild to severe ranges on depression and anxiety scales, indicating the clinical significance of these conditions. Chronic disease burden, functional disability, and corticosteroid treatment may underlie these mental health difficulties. Furthermore, systemic inflammation in BD might contribute to the development of depression and anxiety. Routine psychological assessment in BD clinics should be made as a standard care since the prevalence of the mentioned conditions is high and affects patients’ quality of life and treatment outcomes, as our findings demonstrate that many patients suffer from them and because there is an indication that causes are interrelated and ultimately affect health outcomes suggesting the potential for integrated mental health care.

There are some limitations to this study that need to be considered. First, the marked heterogeneity (I ² > 90%) observed for the pooled estimates of the prevalence of both depression and anxiety reflects substantial variability between studies, which can be attributed to differences in assessment methods, diagnostic criteria, and study populations. This variability makes the findings less generalizable. Second, a variety of self-reported measures and screening instruments were used (i.e., PHQ-9, HADS, DSM-IV), which may lead to measurement bias because some tools can either over- or under-estimate prevalence rates. Third, the cross-sectional design of the majority of studies included in this review limits the ability to portray the causal relationship between BD and psychiatric comorbidities. Furthermore, the use of different diagnostic and screening tools across studies — particularly the inclusion of highly specific instruments like MINI alongside broader screening scales such as PHQ-9 and HADS — limits the validity of direct comparisons between regions and may exaggerate or obscure true geographic differences in mental health burden. Additionally, most included studies did not specify the diagnostic criteria used for Behçet’s disease. Given that classification systems such as the ISG and ICBD differ in sensitivity and organ-specific weighting, variability in patient selection may have influenced the homogeneity of the study populations and, consequently, the mental health outcomes reported. This lack of standardization represents another potential source of heterogeneity and should be considered in future research. Finally, although we did not find evidence of publication bias, the possibility that small studies with null results were underrepresented in our review cannot be ruled out.

There are some major strengths of this study that can shed light on the mental health burden in BD patients. First, it is one of the most comprehensive meta-analyses to date, integrating data from 16 depression and 11 anxiety studies to provide robust pooled prevalence estimates. It is therefore a large sample with greater statistical power and higher reliability of the findings. Second, analyzing studies across different countries, especially in those regions that form the Silk Road where BD is more common [47], should provide important insights on the effects of cultural and healthcare-related factors on BD’s mental health outcomes. Third, by examining subgroup differences (e.g., according to region and assessment tools), the study enhances understanding of factors that underlie heterogeneity. Finally, the fact that no publication bias was detected only strengthens the findings. These strengths emphasize the significance of the current study as it highlights the high prevalence of depression and anxiety in BD patients and the need for integrated mental health care in the management of this complex disease.

## Conclusion and limitation

In conclusion, this systematic review and meta-analysis highlights the significant burden of depression and anxiety in patients with Behçet’s disease (BD), with pooled prevalence rates of 47.44% and 48.48%, respectively. The high heterogeneity observed across studies underscores the variability in prevalence estimates, influenced by differences in assessment tools, geographic regions, and cultural factors. Notably, Egypt reported the highest prevalence of both depression and anxiety, while France had the lowest, suggesting that regional and healthcare-related factors play a critical role in mental health outcomes. The study also emphasizes the importance of using standardized assessment tools, as prevalence rates varied significantly depending on the instrument used. Despite limitations such as cross-sectional designs and potential measurement bias, the findings underscore the need for routine psychological assessments and integrated mental health care in BD management. The high prevalence of psychiatric comorbidities in BD patients, coupled with the chronic and debilitating nature of the disease, calls for a multidisciplinary approach to treatment. Addressing mental health in BD patients is crucial not only for improving quality of life but also for enhancing overall disease outcomes. Future research should focus on longitudinal studies to better understand the causal relationships between BD and psychiatric comorbidities, as well as the development of targeted interventions to address these challenges. There are some limitations to this study that need to be considered. First, the marked heterogeneity (I ² > 90%) observed for the pooled estimates of the prevalence of both depression and anxiety reflects substantial variability between studies, which can be attributed to differences in assessment methods, diagnostic criteria, and study populations. This variability makes the findings less generalizable. Second, a variety of self-reported measures and screening instruments were used (i.e., PHQ-9, HADS, DSM-IV), which may lead to measurement bias because some tools can either over- or under-estimate prevalence rates. Third, the cross-sectional design of the majority of studies included in this review limits the ability to portray the causal relationship between BD and psychiatric comorbidities. Furthermore, the use of different diagnostic and screening tools across studies — particularly the inclusion of highly specific instruments like MINI alongside broader screening scales such as PHQ-9 and HADS — limits the validity of direct comparisons between regions and may exaggerate or obscure true geographic differences in mental health burden. Additionally, most included studies did not specify the diagnostic criteria used for Behçet’s disease. Given that classification systems such as the ISG and ICBD differ in sensitivity and organ-specific weighting, variability in patient selection may have influenced the homogeneity of the study populations and, consequently, the mental health outcomes reported. This lack of standardization represents another potential source of heterogeneity and should be considered in future research. Another important limitation is that only a small number of the included studies incorporated healthy control groups or comparator populations. Although some studies reported no significant differences in depression and anxiety rates between patients with Behçet’s disease and healthy individuals, most studies evaluated psychiatric symptoms exclusively within BD populations. Consequently, it remains difficult to determine whether the observed prevalence truly exceeds that expected in the general population or among individuals with other chronic diseases. Future research should prioritize well-designed controlled studies to better characterize the relative burden of psychiatric comorbidities in patients with BD. Finally, although we did not find evidence of publication bias, the possibility that small studies with null results were underrepresented in our review cannot be ruled out.Additionally, the protocol for this review was not prospectively registered in PROSPERO.

## Supplementary Information

Below is the link to the electronic supplementary material.

Supplementary Material 1

## Data Availability

All data generated or analyzed during this study are included in this published article [and its supplementary information files].
