# Supplementary material for: Prevalence of depression and anxiety among patients with Behçet’s disease: a systematic review and meta-analysis
Source: Adv Rheumatol. 2026 Oct 29;66:62. doi: 10.1186/s42358-026-00591-7 (PMC13721388; doi:10.1186/s42358-026-00591-7)
Supplement: Supplementary file 1 [file 42358_2026_591_MOESM1_ESM.docx]

PubMed search query:

("Behcet Syndrome"[Mesh] OR "behcet*"[tiab] OR "behcet's"[tiab] OR "Adamantiades-Behcet"[tiab] OR "Silk Road disease"[tiab])

AND

("Depression"[Mesh] OR "Depressive Disorder"[Mesh] OR "depress*"[tiab] OR "major depressive disorder"[tiab] OR "mood disorder*"[tiab] OR "Anxiety"[Mesh] OR "Anxiety Disorders"[Mesh] OR "anxiet*"[tiab] OR "anxious*"[tiab] OR "panic disorder*"[tiab])

Embase search query:

('behcet disease'/exp OR behcet*:ti,ab,kw OR 'adamantiades behcet':ti,ab,kw OR 'silk road disease':ti,ab,kw)

AND

('depression'/exp OR depress*:ti,ab,kw OR 'mood disorder'/exp OR 'mood disorder*':ti,ab,kw OR 'anxiety'/exp OR anxiet*:ti,ab,kw OR 'anxious*':ti,ab,kw OR 'anxiety disorder'/exp OR 'panic disorder*':ti,ab,kw)

Scopus search query:

TITLE-ABS-KEY ( behcet* OR {Behcet's disease} OR {Behcet syndrome} OR "Adamantiades-Behcet*" OR "Adamantiades Behcet*" OR "Silk Road disease" )

AND

TITLE-ABS-KEY ( depress* OR {major depressive disorder} OR "mood disorder*" OR anxiet* OR anxious* OR {panic disorder} OR "psychological distress*" OR "psychiatric comorbidity" OR "mental disorder*" OR "mental health" OR "psychiatric symptom*" )

**Supp. Fig. 1 — Result of subgroup analysis for depression.**

**Supp. Fig. 2 — Result of subgroup analysis for anxiety.**

| ID | Author | 1 | 2 | 3 | 4 | 5 | 6 | 7 | 8 | 9 | Quality Score |
| --- | --- | --- | --- | --- | --- | --- | --- | --- | --- | --- | --- |
| 1 | RECEP DURSUN | Yes | Yes | Yes | Yes | Yes | Yes | Yes | Yes | Yes | include |
| 2 | Mukadder Koçak | NO | Yes | Yes | Yes | Yes | Yes | Yes | Yes | Yes | include |
| 3 | Aysun Kalenderoglu | NO | Yes | Yes | Yes | Yes | Yes | Yes | Yes | Yes | include |
| 4 | Birkan ILHAN | Yes | Yes | Yes | Yes | Yes | Yes | Yes | Yes | Yes | include |
| 5 | Ozcan Hiz | NO | Yes | Yes | Yes | Yes | Yes | Yes | Yes | Yes | include |
| 6 | I.G. Gu¨ l | Yes | Yes | Yes | Yes | Yes | Yes | Yes | Yes | Yes | include |
| 7 | Sevinc Can Sandikci | Yes | Yes | Yes | Yes | Yes | Yes | Yes | Yes | Yes | include |
| 8 | Hilal Eser-Öztürk | NO | Yes | Yes | Yes | Yes | Unclear | Yes | Yes | Unclear | include |
| 9 | Elif Gur Kabul | Yes | Yes | Yes | Yes | Yes | Yes | Yes | Yes | Yes | include |
| 10 | Murat Oklar | yes | yes | yes | yes | yes | yes | yes | yes | yes | include |
| 11 | Philip I. Murray | yes | unclear | yes | unclear | yes | yes | yes | yes | unclear | include |
| 12 | Carlos Eduardo Garcez Teixeira | yes | yes | yes | yes | yes | yes | yes | yes | no | include |
| 13 | Hania Zayed | yes | unclear | yes | yes | yes | yes | yes | yes | no | include |
| 14 | Levent YAZMALAR | yes | yes | yes | yes | yes | yes | yes | yes | no | include |
| 15 | Fanny Urbain | yes | not applicable | no | yes | yes | yes | yes | yes | yes | include |
| 16 | Sebastian‑Jonas Saur | yes | yes | yes | yes | yes | yes | yes | yes | no | include |
| 17 | bar pitaro alter | yes | yes | yes | yes | yes | unclear | unclear | yes | yes | include |

**Quality assessment table (based on JBI scale)**
